# Low-Fidelity Polymerases of Alphaviruses Recombine at Higher Rates To Overproduce Defective Interfering Particles

**DOI:** 10.1128/JVI.02921-15

**Published:** 2016-02-11

**Authors:** Enzo Z. Poirier, Bryan C. Mounce, Kathryn Rozen-Gagnon, Peter Jan Hooikaas, Kenneth A. Stapleford, Gonzalo Moratorio, Marco Vignuzzi

**Affiliations:** a Institut Pasteur, Centre National de la Recherche Scientifique, UMR 3569, Paris, France; b University of Paris Diderot, Sorbonne Paris Cité, Cellule Pasteur, Paris, France

## Abstract

Low-fidelity RNA-dependent RNA polymerases for many RNA virus mutators have been shown to confer attenuated phenotypes, presumably due to increased mutation rates. Additionally, for many RNA viruses, replication to high titers results in the production of defective interfering particles (DIs) that also attenuate infection. We hypothesized that fidelity, recombination, and DI production are tightly linked. We show that a Sindbis virus mutator replicating at a high multiplicity of infection manifests an earlier and greater accumulation of DIs than its wild-type counterpart. The isolated DIs interfere with the replication of full-length virus in a dose-dependent manner. Importantly, the ability of the mutator virus to overproduce DIs could be linked to an increased recombination frequency. These data confirm that RNA-dependent RNA polymerase fidelity and recombination are inversely correlated for this mutator. Our findings suggest that defective interference resulting from higher recombination rates may be more detrimental to RNA virus mutators than the increase in mutational burden.

**IMPORTANCE** Replication, adaptation, and evolution of RNA viruses rely in large part on their low-fidelity RNA-dependent RNA polymerase. Viruses artificially modified in their polymerases to decrease fidelity (mutator viruses) are attenuated *in vivo*, demonstrating the important role of fidelity in viral fitness. However, attenuation was attributed solely to the modification of the viral mutation rate and the accumulation of detrimental point mutations. In this work, we described an additional phenotype of mutator viruses: an increased recombination rate leading to defective interfering particle (DI) overproduction. Because DIs are known for their inhibitory effect on viral replication, our work suggests that fidelity variants may be attenuated *in vivo* via several mechanisms. This has important implications in the development of fidelity variants as live attenuated vaccine strains.

## INTRODUCTION

During an infection, RNA viruses generate extensive diversity due to their high mutation rates (10^−4^ mutations per nucleotide copied), which is largely attributable to the error rate of the viral RNA-dependent RNA polymerase (RdRp) (1, 2). This past decade, the engineering of RNA viruses of various families with altered mutation rates has shown that single substitutions can significantly alter polymerase activity and fidelity. Among these variants, RdRp mutators present point mutations in the viral polymerase that lead to increased mutation rates compared to those of the wild-type (WT) virus. When mutator variants are introduced *in vivo* in various animal models, the majority are significantly attenuated (3–7). For example, RdRp mutator variants of chikungunya virus (CHIKV) and Sindbis virus (SINV) are attenuated in fruit flies and mice (4). The attenuation observed *in vivo* has been directly correlated with the increased mutation rates that would presumably result in higher frequencies of lethal mutations. Indeed, mutator strains of RNA viruses present mutation frequency profiles similar to those of wild-type-like viruses whose mutation rates are extrinsically increased by treatment with RNA mutagens in studies evaluating lethal mutagenesis as an antiviral strategy (8). However, while many determinants that alter RdRp fidelity have been identified, the enzymatic mechanisms or dynamics by which this occurs are not well elucidated, and how decreasing fidelity may affect other polymerase activities is unclear (9, 10).

Another curious feature of RNA virus replication is the production of truncated genomes and defective interfering particles (DIs). First reported in 1947 by von Magnus and later explored by Huang and Holland during the 70s and the 80s, DI production is now described in almost every RNA virus family (11–19). DIs are truncated forms of viral genomes that accumulate during replication, especially during passage in cell culture at a high multiplicity of infection (MOI). Because DIs lack one or several parts of coding/noncoding sequence, they highjack full-length virus proteins for replication, packaging, and transmission. By doing so, they interfere with the replication of the full-length virus (20). In addition to this interference, DIs are implicated in a preferential activation of type I interferon *in vivo*, via detection by the cytoplasmic sensors RIG-I and MDA5 (21–23). They also enhance dendritic cell maturation (24). The ability to recombine in a homologous or nonhomologous manner, an important driver of viral evolution, is a well-known feature of RNA viruses (25). This process relies on a copy choice mechanism, when the viral RdRp switches templates during RNA synthesis while pursuing elongation of the nascent RNA. DI formation corresponds, in this context, to a switch between two noncolinear fragments of the viral genome, which could equally happen between two separated viral RNAs (intermolecular) or on one viral genome (intramolecular) (20, 25). A second mechanism of DI formation relies on nonreplicative recombination when fragments of viral genomes can be religated together independently of RdRp activity (26, 27). However, such events are thought to be rare enough so that the vast majority of DIs arise from copy choice recombination (25).

In this work, we characterized the behavior of an RdRp mutator of Sindbis virus (SINV) of the Togaviridae family. We first assessed the ability of an SINV mutator, termed SINV-G (SINV with a C482G mutation, SINV-C482G), to produce DI particles during passage at a high multiplicity of infection (MOI). We monitored the accumulation of DIs throughout the passages and showed that SINV-G accumulated DIs at a higher rate. DIs produced by SINV-G were further implicated in interference with full-length virus replication. Strikingly, we observed an important increase in the recombination rate of this mutator. Our findings demonstrate that along with increased error rate, higher recombination rates are a general characteristic of the RdRp mutator. Furthermore, the higher recombination rates lead to increased DI production, which may be a previously overlooked and main contributor to mutator *in vivo* attenuation.

## MATERIALS AND METHODS

### Viruses and cells.

Mammalian cell lines Vero and BHK-21 were maintained in Dulbecco's modified Eagle's medium (DMEM; Gibco) supplemented with 10% newborn calf serum (NCS; Gibco) and 1% penicillin-streptomycin (P-S; Sigma) at 37°C with 5% CO_2_. Sindbis viruses were generated from the pTR339 infectious clone and were linearized with XhoI (28). Linearized products were then purified by phenol-chloroform extraction and subsequently used for *in vitro* transcription of viral RNAs using an SP6 mMESSAGE mMACHINE kit (Ambion). RNAs were purified by phenol-chloroform extraction, quantified, diluted to 1 μg/μl, and stored at −80°C. To generate virus stocks, BHK-21 cells were transfected with viral RNA as follows: cells were trypsinized, washed twice with ice-cold phosphate-buffered saline (PBS), and resuspended at a concentration of 2 × 10^7^ cells/ml in ice-cold PBS. Cells (0.390 ml) were mixed with 10 μg of *in vitro*-transcribed (IVT) viral RNA, placed in a 2-mm cuvette, and electroporated at 1.2 kV and 25 μF with infinite Ω in an XCell Gene Pulser (Bio-Rad). Following electroporation, cells were then plated in a T-25 flask. After 48 h, supernatant was collected and titrated by plaque assay. The virus was passaged over a 70 to 80% confluent monolayer of BHK-21 cells in order to generate virus stocks. Virus supernatant was aliquoted at 24 h postinfection and stored at −80°C. Mutator stocks were resequenced at the RdRp mutation position to check for reversion.

### Virus titer by plaque assay.

Twelve-well plates were seeded with 400,000 Vero cells per well the day before. The following day, 10-fold serial dilutions of each virus in DMEM were incubated on the cells' monolayers for 1 h at 37°C. Following incubation, cells were overlaid with DMEM containing 2% NCS and 0.8% agarose, and plates were incubated at 37°C for 72 h. The cells were then fixed with 4% formalin for 1 h, and plaques were visualized by the addition of crystal violet.

### Growth curve and passaging.

BHK-21 cells were infected for 1 h at 37°C at the MOI indicated in the text and figure legends. Virus was removed by extensive PBS washing, and then supernatant was collected at various time points postinfection. Passages were carried out in a total of 2 ml of supernatant at the MOI indicated in the text and figure legends. If the viral titer was too low to allow such an MOI, a fixed amount of 1.5 ml of supernatant was used to infect the next passage.

### Northern blot analysis.

RNA from infected cells or virus suspension was extracted using TRIzol reagent (Invitrogen). RNA samples were prepared using NorthernMax formaldehyde loading dye (Ambion) and 1 μl of ethidium bromide and then heated to 65°C for 20 min. Samples were then separated on a 1.2% low-electroendosmosis (LE) agarose (Lonza) gel containing 1× morpholinepropanesulfonic acid (MOPS) running buffer (Ambion) and 6.7% formaldehyde. RNA was transferred onto nitrocellulose membrane overnight and then cross-linked by UV irradiation (UVP), and the membrane was blocked for 1 h at 68°C in ULTRAhyb ultrasensitive hybridization buffer (Ambion). RNA probes complementary to positive-strand RNA were then synthesized using a MAXIscript SP6 or T7 *in vitro* transcription kit (Ambion) and labeled with ^32^P. After removal of unincorporated nucleotides using Illustra MicroSpin S200 HR columns, probes were hybridized to membranes overnight at 68°C. Membranes were then washed three times with washing buffer (0.1× SSC–0.1% SDS in autoclaved water; 1× SSC is 0.15 M NaCl plus 0.015 M sodium citrate) at 68°C for 20 min and then imaged using Amersham Hyperfilm MP autoradiography film (GE Healthcare).

### RNase A treatment and purification by ultracentrifugation.

Viral supernatant was centrifuge at 1,200 × *g* for 5 min to remove dead cells and then treated with 10 μg/ml of RNase A (Thermo Scientific) at room temperature for 1 h. Three milliliters of 20% sucrose diluted in PBS was poured in a 12.5-ml open-top centrifuge tube (Beckman Coulter) and then overlaid with 8 ml of viral supernatant. Tubes were centrifuged at 107,000 × *g* for 2 h at 4°C using an SW41 Ti rotor in an Optima XPN-100 ultracentrifuge (Beckman Coulter). Supernatant was removed, and the purified virus pellet was resuspended in 1 ml of PBS and stored at −80°C.

### Reverse transcription-PCR (RT-PCR), quantitative reverse transcription-PCR (qRT-PCR), PCR, and gel extraction.

RNA samples were retrotranscribed into cDNA using a Maxima H Minus First Strand cDNA synthesis kit (Thermo Scientific) with oligo(dT), according to the manufacturer's instructions. To detect Dg (a defective genome in SINV-G), PCR was performed using Phusion polymerase (Thermo Scientific) and the primer pair 414F (5′-AAGGATCTCCGGACCGTACT-3′) and 11634R (5′-ATTATGCACCACGCTTCCTCAGA-3′). To obtain the sequence of the Dg, the band was gel extracted using a QIAquick gel extraction kit (Qiagen) and then submitted to a second PCR with the same primers. Sequences were obtained from GATC Biotech. For quantitative PCR (qPCR), cDNA was mixed with power SYBR green PCR master mix (Applied Biosystems) and the following primer pairs: 4357F (5′-AAAACGCCTACCATGCAGTG-3′) and 4454R (5′-TTTTCCGGCTGCGTAAATGC-3′) for full-length genomes, 1634F (5′-TGCGAAGTGGAGGGGCTCC-3′) and 10565R (5′-TGAAATTGGTCCAGCTATGACTTT-3′) for Dg, and 1725F (5′-GCAAATGACCGTATGATCGGA-3′) and 11297R (5′-AAACAGCCAACTCCATGATG-3′) for Dwt (a defective genome in SINV-WT). A StepOne Plus real-time PCR system machine (Applied Biosystems) was used according to the manufacturer's instructions.

### Transfection and stable cell lines.

293T cells were transfected with a mix of RNA totalizing 0.5 μg using Jetprime reagent, according to the manufacturer's instructions. To establish stable cell lines, pcDNA3.1(+) plasmid (Life Technologies) was engineered to express the construct of interest, linearized, and then transfected into BHK-21 cells. Stable cell lines were isolated by endpoint dilution according to the manufacturer's instructions.

### Statistical tests.

Unless indicated otherwise, each experiment was performed in technical triplicate and repeated three times (biological triplicate). All tests were performed using Microsoft Excel and GraphPad Prism. *P* values of >0.05 were considered nonsignificant.

## RESULTS

### SINV mutator RdRp presents altered replication kinetics at high-MOI passage in cell culture.

**FIG 1:**
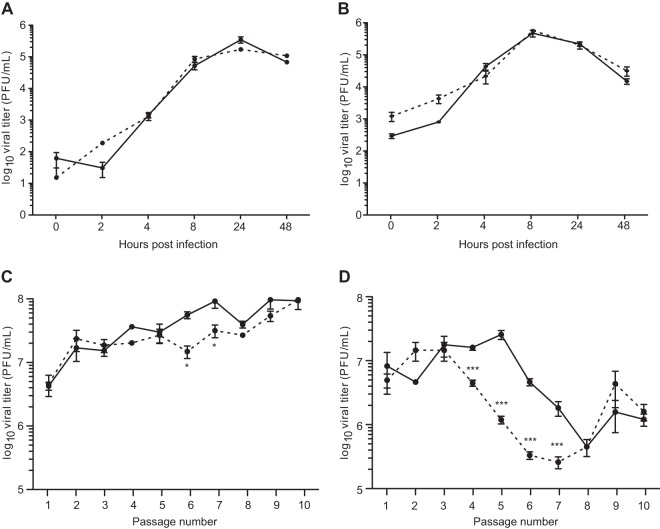
SINV mutator RdRp presents altered replication kinetics at high-MOI passage in cell culture. (A and B) Growth curve of SINV-WT (solid line) and SINV-G (dashed line) at MOIs of 1 (A) and 25 (B). Mean values and standard errors of the means are shown (*n* = 3). Data were analyzed by two-way analysis of variance with Bonferroni's posttest (no significant differences were determined). (C and D) SINV-WT (solid line) and SINV-G (dashed line) were passaged 10 times in BHK-21 cells at MOIs of 1 (C) or 25 (D), and virus titers were measured by plaque assay. Mean values and standard errors of the means are shown (*n* = 3). Data were analyzed by two-way analysis of variance with a Bonferroni posttest (*, *P* < 0.05; ***, *P* < 0.001).

While the SINV mutator (SINV-G) had wild-type-like growth kinetics in one-step growth curves at low (Fig. 1A) and high (Fig. 1B) multiplicities of infection (MOIs), we further assessed its activity over extended passage. We thus passaged the SINV wild-type (SINV-WT) and SINV-G at a low (MOI of 1) and high (MOI of 25) MOI over 10 passages and assessed viral titers. Sequencing confirmed that the SINV-C482G mutation was stable throughout the passages. While SINV-WT and SINV-G presented similar titers and kinetics at low-MOI passage (Fig. 1C), SINV-G significantly dropped in titer two passages earlier than SINV-WT at a high MOI (Fig. 1D). Indeed, DIs accumulate and trigger a drop in viral titer via defective interference during high-MOI passages in cell culture (20). These data suggest that the SINV-G mutator may be producing DI particles more rapidly or in greater numbers than wild-type virus. These DIs might be viral genomes bearing deleterious mutations, which could accumulate to a greater extent during mutator virus passages. Alternatively, these DIs could be internally deleted genomes.

### SINV-G mutator RdRp produces more defective particles.

**FIG 2:**
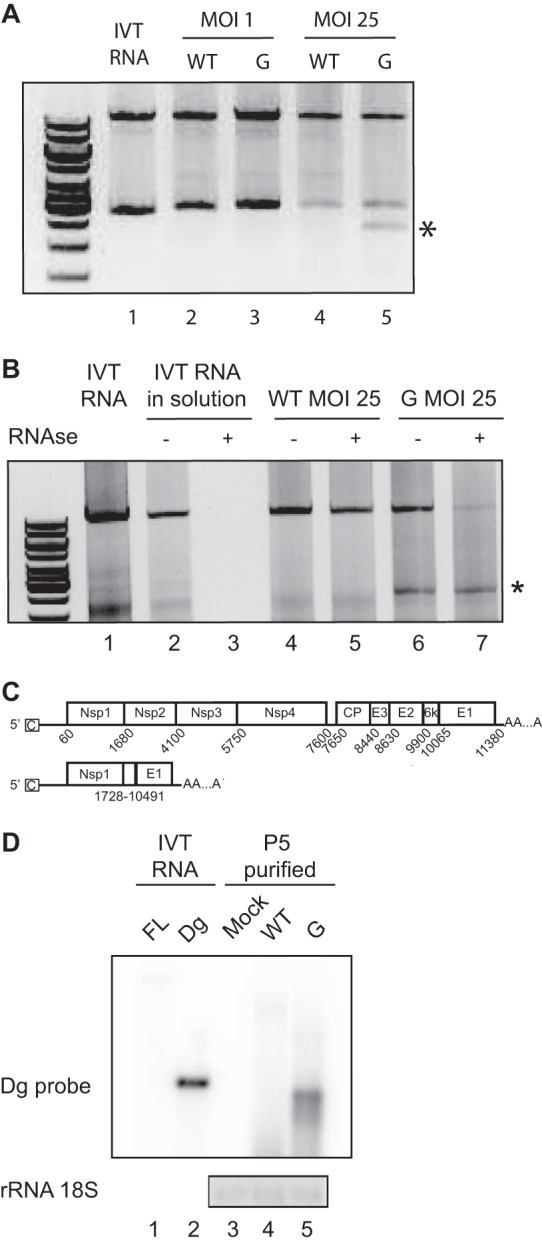
Defectives particles can be detected during SINV-G infection. (A) Detection of SINV-WT (WT) and SINV-G (G) genomic sequences by RT-PCR after 24 h of infection at an MOI of 1 or 25. *In vitro*-transcribed RNA (lane 1) was used as a control. The top band corresponds to the full-length genome. An extra low-molecular-weight band is indicated by an asterisk. (B) Viral supernatants from a 24-h infection with SINV-WT (lanes 4 and 5) or SINV-G (lanes 6 and 7) were either untreated or treated with RNase A (−, absence; +, presence) and then subjected to RT-PCR as described for panel A. *In vitro*-transcribed RNA (lane 1) was used as an RT-PCR control. IVT RNAs incubated in solution (lane 2) and treated with RNase A (lane 3) were used as RNase controls. (C) Schematic of SINV full-length genome and the defective genome, Dg. Schematic shows the 5′ and 3′ untranslated regions with cap (C) and poly(A) tail and the intergenic region containing the subgenomic promoter (arrow) for structural proteins. Each protein-coding gene is depicted as a separate box, with positions of start and stop nucleotides numbered below. The deleted nucleotides are numbered below the Dg scheme. (D) Five undiluted passages were performed on BHK-21 cells with SINV-WT (lane 4) or SINV-G (lane 5) virus, or cells were mock infected (lane 3). Progeny virions were treated with RNase and purified by ultracentrifugation. Viral RNA was probed by Northern blotting using a probe specifically targeting the Dg breakpoint. *In vitro*-transcribed (IVT) RNAs corresponding to full-length (FL) and Dg genomes, not treated with RNase, were used as positive controls for detection (lanes 1 and 2).

To address this hypothesis and isolate the putative DIs, we reasoned that the DI's ability to interfere relies on its capacity to be replicated, encapsidated, and transmitted during passages. Consequently, DIs should conserve replication signals (carried by the 5′ and 3′ ends of the genome), as well as the encapsidation signal (in the nsP1 coding sequence). We set up an RT-PCR with PCR primers located at the 5′ and 3′ ends of the genome that should anneal to both the full-length genome and the DIs. When performed on *in vitro*-transcribed (IVT) RNA, this PCR yielded an 11-kb band, signaling the presence of the full-length genome (Fig. 2A, lane 1). As expected, full-length genomes were amplified in the supernatants of cells infected with SINV-WT or SINV-G at MOIs of 1 and 25 (Fig. 2A, lanes 2 to 5). In contrast, an extra low-molecular-weight band was amplified solely in the sample infected with SINV-G at an MOI of 25 (Fig. 2A, lane 5). To verify that this putative defective genome was encapsidated in viral particles, we subjected virus-containing supernatants to RNase treatment. Following such treatment, IVT RNA in solution was completely degraded and was not amplified by PCR (Fig. 2B, lane 3). The low-molecular-weight band detected in the SINV-G high-MOI samples was still present after RNase treatment (Fig. 2B, lane 7). We next purified and sequenced this band, which yielded a viral genome with a deletion of an 8.7-kb span of mostly coding sequences (Fig. 2C). As expected, this defective genome, named Dg, retained the encapsidation signal and the 5′ and 3′ untranslated regions. We further showed the production of Dg by Northern blotting. SINV-WT and -G were submitted to five undiluted passages and then concentrated by ultracentrifugation on sucrose cushion after RNase treatment. We extracted RNA from this pure virion population and labeled it with a probe overlapping the Dg breakpoint. As expected, IVT RNA corresponding to the Dg sequence was strongly detected while the full-length genome IVT RNA was barely detected (Fig. 2D, lanes 1 and 2). While no bands were detected in uninfected cells (Fig. 2D, lane 3) or SINV-WT samples (Fig. 2D, lane 4), SINV-G infection showed a clear accumulation of Dg (Fig. 2D, lane 5). In addition to Dg, which appears to be the main defective genome accumulated during the passages, we were able to recover other truncated genome sequences using a similar cloning technique (data not shown).

**FIG 3:**
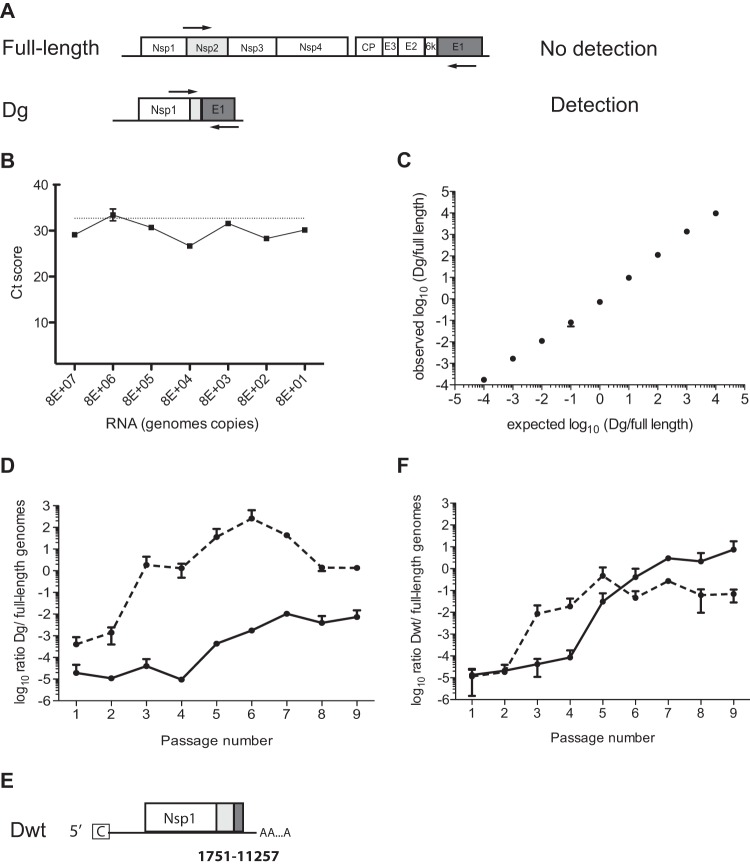
SINV-G mutator RdRp produces more defective particles. (A) In order to detect only Dg in the sample, primers 1634F and 10565R were chosen on each side of the breakpoint (arrows). (B) Increasing amounts of *in vitro*-transcribed full-length RNA was assessed by qRT-PCR using these Dg primers. Regardless of the amount, the signal remained at the same level as that of the water-only control (dashed line). *C_t_*, cycle threshold. (C) *In vitro*-transcribed RNAs of the full-length genome and Dg were mixed at different ratios and then measured by qRT-PCR using a primer pair targeting only the full-length genome and the aforementioned primer pair detecting only the Dg genome. The measured ratios (observed) were compared to input ratios (expected). Mean values and standard errors of the means are indicated (*n* = 3). (D) The ratio of the Dg genome to full-length genome was quantified by qRT-PCR in samples of SINV-WT (solid line) and SINV-G (dashed line) passaged at an MOI of 25, as shown in Fig. 1D. Mean values and standard errors of the means are shown (*n* = 3). (E) Schematic of defective genome Dwt, as described in the legend of Fig. 2C. (F) Ratios of Dwt genomes to full-length genomes were quantified by qRT-PCR in samples passaged at an MOI of 25 of SINV-WT (solid line) and SINV-G (dashed line), as shown in Fig. 1D. Mean values and standard errors of the means are shown (*n* = 3).

We then used qPCR to analyze the accumulation of Dg during high-MOI passage. A first primer pair located in the deleted region of Dg was used to measure the amount of full-length genomes. Independently, primers located at the 5′ and 3′ ends of the Dg breakpoint allowed the specific detection of Dg since the distance between these two primers on a full-length genome was too great to permit full-length genome detection. (Fig. 3A to C). Dg quickly accumulated during high-MOI passage of SINV-G (Fig. 3D), reaching more than 100 copies per full-length genome by passage 6. The increasing ratio of Dg to genomes inversely correlated with virus titers throughout the passage series (Fig. 1D). SINV-WT also showed an accumulation, albeit slower, of Dg throughout the passages, indicating that the production of this specific Dg does not represent a unique or artificial feature of the SINV-G mutator. However, the moderate accumulation of Dg for SINV-WT could not explain the drop in titers observed after passage 6 (Fig. 1D). We thus applied an identical methodology to isolate yet another defective genome in SINV-WT passages, named Dwt (Fig. 3E). Despite being 600 nucleotides shorter, Dwt presented the same characteristics as Dg, with a 9.5-kb internal deletion. Of note, the primers used to initially detect Dg by qRT-PCR could not detect Dwt due to its larger deletion. As expected, Dwt accumulated during SINV-WT passage, reaching 50 copies per full-length genome (Fig. 3F), with kinetics that similarly mirrored the drop in SINV-WT titer during passage. As for Dg, SINV-G showed a stronger accumulation of Dwt in the earlier passages (Fig. 3F). Taken together, these data indicate that SINV-G more rapidly produced and more significantly accumulated naturally occurring defective genomes.

### SINV defective genomes interfere with full-length virus replication.

**FIG 4:**
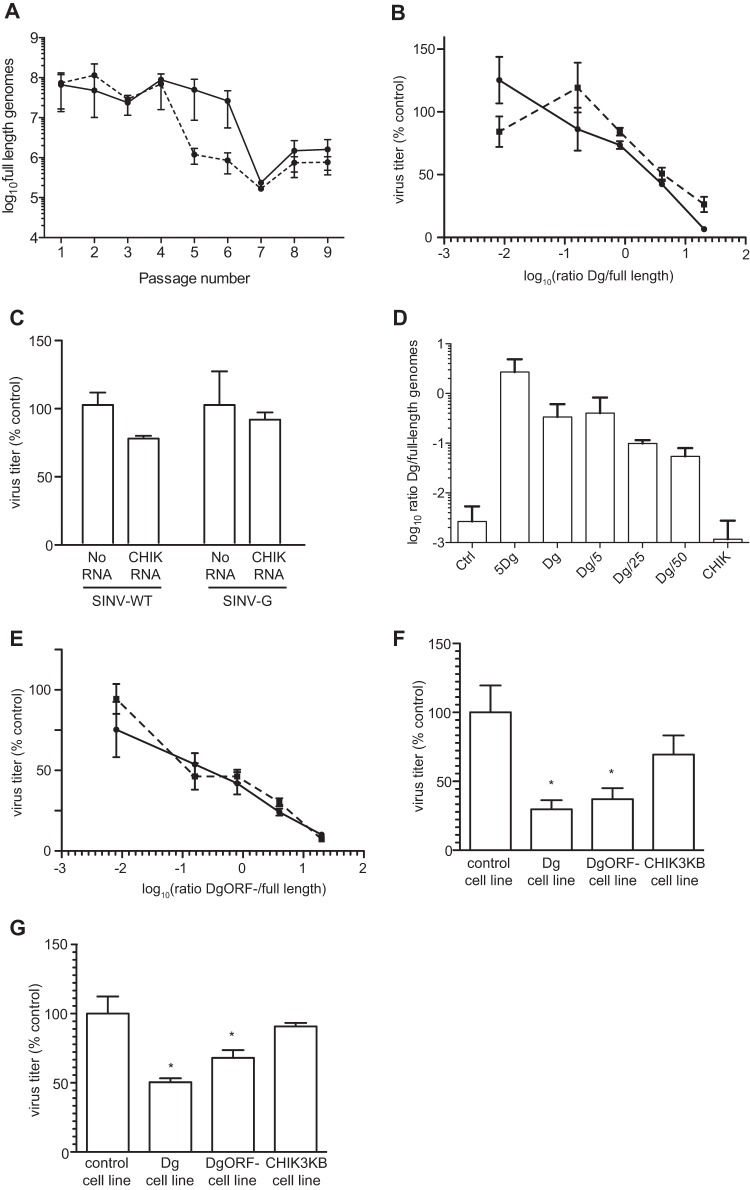
SINV defective genomes interfere with full-length virus replication. (A) The number of full-length genomes in high-MOI passages of the SINV-WT (solid line) and SINV-G (dashed line) was assessed by qRT-PCR. Means and standard errors of the means are shown (*n* = 3). (B) 293T cells were transfected with a fixed amount of full-length SINV-WT (solid line) or SINV-G (dashed line) RNA and an increasing amount of Dg RNA. Titer was measured by plaque assay at 24 h posttransfection. Means and standard errors of the means are shown (*n* = 3). (C) Chikungunya virus replication-defective RNA (CHIK RNA) does not significantly interfere with SINV-WT or SINV-G virus replication compared to replication in infected cells with no additional RNA added (No RNA). Means and standard errors of the means are shown (*n* = 3; no significant differences were detected by a two-tailed student *t* test). (D) Viral supernatant from the transfection presented in panel B was subjected to RNase A treatment before qRT-PCR to measure the Dg/full-length genome ratio as previously described. The *x* axis indicates the ratio of Dg/full-length RNA initially transfected. Means and standard errors of the means are shown (*n* = 3). (E) 293T cells were transfected with a fixed amount of full-length SINV-WT (solid line) or SINV-G (dashed line) RNA and an increasing amount of Dg RNA mutated in its nsP1 starting codon (DgORF^−^). Titer was measured by plaque assay at 24 h posttransfection. Means and standard errors of the means are shown (*n* = 3). (F and G) Stable cell lines expressing Dg, DgORF^−^, or CHIK3KB were infected with either SINV-WT (F) or SINV-G (G), and titers were measured at 16 h postinfection. Means and standard errors of the means are shown (*n* = 3; *, *P* < 0.05, for sample values compared to those of the wild-type, by one-way analysis of variance with a Bonferroni posttest).

One explanation for the earlier drop in titer of SINV-G during passage could be the production of a greater number of full-length genomes, which could result in an increased accumulation of genomes spiked with deleterious mutations, independently of the generation of truncated genomes. This hypothesis is supported by the fact that mutator viruses have a lower specific infectivity and higher elongation rate (4, 10). However, similar accumulations of full-length genomes were detected from passages 1 to 4 for both viruses (Fig. 4A), ruling out this hypothesis. On the other hand, the accumulation of Dg and Dwt throughout passages could explain the drop in titer if these defective genomes truly interfered with full-length genome replication, as is expected of DIs. To address this possibility, we cloned Dg into a plasmid, and Dg RNA was synthetized *in vitro*. We cotransfected 293T cells with a fixed amount of full-length RNA of either SINV-WT or SINV-G and an increasing amount of Dg RNA and assessed viral titers after 24 h. As Dg RNA amounts increased for both SINV-WT and -G viruses, virus titers dropped to 10% of the control value for a 5:1 Dg/full-length virus ratio, showing that Dg indeed interfered with full-length viral replication (Fig. 4B). As a negative control, we used a full-length chikungunya virus RNA unable to replicate instead of the Dg RNA (Fig. 4C). In addition, we detected Dg accumulation in viral supernatants of transfected cells (Fig. 4D). Recently, a truncated protein expressed from a DI of influenza A virus PB2 protein was implicated in the attenuation of the full-length virus *in vitro* (29). Because Dg and Dwt conserved an intact 5′ end, they theoretically could be translated and yield a full nsP1 protein as well as a chimeric nsP2-E1 protein. To rule out the participation of such protein products in the attenuation of full-length SINV, we engineered a Dg mutated in its start codon (DgORF^−^, where ORF is open reading frame). When cotransfected with full-length RNA, DgORF^−^ inhibited the accumulation of full-length virus in a similar fashion (Fig. 4E). As an alternative approach, we engineered stable BHK cell lines expressing Dg, DgORF^−^, or the first 3 kb of the CHIKV genome (CHIK3KB). qPCR results confirmed that each transcript was robustly expressed in each stable cell line (data not shown). When infected for 16 h with SINV-WT (Fig. 4F) or SINV-G (Fig. 4G), BHK cells expressing either Dg or DgORF^−^ yielded 2 to 3 times less virus than control cell lines or cells stably expressing CHIK3KB RNA.

### RdRp mutators have higher recombination rates than wild-type virus.

**FIG 5:**
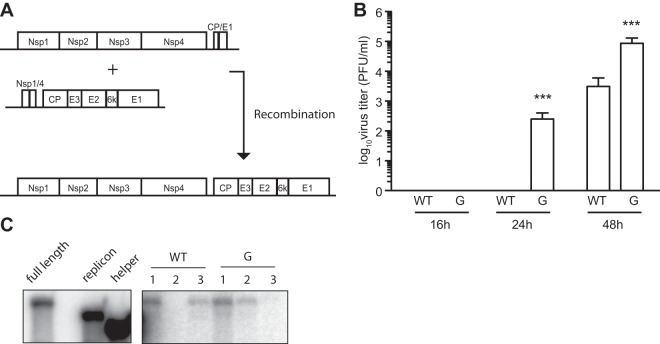
RdRp mutators have higher recombination rates than wild-type virus. (A) Schematic of the recombination experiment: BHK-21 cells were cotransfected with an equal amount of replicon RNA and helper RNA. Titer was assessed by plaque assay. (B) BHK-21 cells were cotransfected with SINV-helper and with replicon-WT or replicon-G. At 16, 24, and 48 h posttransfection, progeny viruses arising from recombination were quantified by plaque assay. Mean values and standard errors of the means are shown (*n* = 3; ***, *P* < 0.001, by a two-tailed student *t* test). (C) BHK-21 cells were infected with progeny collected from the recombination experiment presented in panel B and probed by Northern blotting using a probe able to recognize full-length, replicon, and helper genomes.

DI production relies on an accident of replication when the viral replication complex “jumps” from one part of the genome to another, distant one. Recombination might thus be a driver of DI production. To compare the recombination kinetics of SINV-WT and SINV-G, we electroporated BHK-21 cells with two segments of RNA, each of which could not generate viable progeny alone: one coding for the four proteins of the replication complex (replicon-WT or replicon-G) and the other coding for the structural proteins (Fig. 5A). We then measured the number of viable, full-length genomes that arose from recombination. Interestingly, we detected full-length virus as soon as 24 h postelectroporation in supernatant of replicon-G-electroporated cells (Fig. 5B). At 48 h postelectroporation, replicon-G yielded 10 times more infectious virus than its wild-type counterpart. To verify that plaques truly corresponded to full-length virus, naive cells were infected with viral supernatant from these WT or G electroporations. After 16 h, we extracted RNA from cells and probed viral genomes by Northern blotting (Fig. 5C). As expected, full-length virus was detected from these infections. Overall, these data demonstrate that the RdRp point mutation conferring mutator status to SINV-G also confers a higher recombination rate.

## DISCUSSION

Over the past decade, isolation of RdRp mutators of several viral families and their subsequent attenuation *in vivo* led to the conclusion that fidelity is a critical parameter of virus fitness. In this work, we characterized an additional feature of RNA virus RdRp mutators: their ability to recombine at a higher rate than wild-type viruses. Recombination rates are believed to depend on parameters such as genome organization and specificities of the virus life cycle and replication. For example, high processivity of the RNA virus RdRp is linked to the production of DIs through the copy choice mechanism (25). A higher processivity during replication could explain both characteristics of the SINV mutator: the increased mutation rate (less time to discriminate between different nucleotides) and the formation of DIs (more probable jumps from one part of the genome to another). This hypothesis is supported in a recent report correlating mutator phenotype and elongation rates for mutants of picornaviruses, a correlation particularly robust for mutants of the palm domain (10). Structural modeling of CHIKV RdRp localized residue 483 conserved in SINV as residue 482 in the same palm domain (4).

In this work, we monitored the evolution of SINV-WT and SINV-G titers throughout 10 passages at a high MOI in cell culture. The mutator SINV-G displayed an earlier drop in titer than its wild-type counterpart (Fig. 1D). To explain this drop, we could invoke the accumulation of full-length genomes bearing deleterious point mutations. Indeed, when cells persistently infected with lymphocytic choriomeningitis virus (LCMV) are treated with a mutagenic compound targeting the viral RdRp, viral extinction is observed through accumulation of such deleterious, point-mutated viral genomes (30). However, mutagenic treatment of LCMV led to a maximum increase in mutation rate of 10-fold while SINV-G possesses a 2-fold-higher mutation rate than the wild-type virus (4). Moreover, SINV-WT and SINV-G display similar numbers of full-length genomes in the early passages (Fig. 4A). Thus, accumulation of deleterious full-length genomes is unlikely the reason for a drop in titer throughout the passages. On the other hand, we monitored the accumulation of two main DIs, Dg and Dwt, during high-MOI passage of SINV-WT and SINV-G. These truncated genomes dramatically accumulated, reaching more than 100 copies of Dg per full-length genome. We further showed that these RNA species interfere with the replication of full-length RNA and ruled out the involvement of any protein product. While the presented data do not rule out a possible involvement of nonreplicative recombination in Dg and Dwt formation, the fact that copy choice recombination was described to be the main mechanism of DI formation (20, 25), as well as the increased recombination rate of SINV-G, would tend to incriminate the copy choice mechanism in Dg and Dwt formation. Moreover, because SINV-G recombines at a higher rate, we can assume that its replication produces a diverse cloud of defective genomes, heterogeneous in size and breakpoint sites. Only a small portion of these would be transmissible to other cells as they require both signals for replication (5′ and 3′ ends of the genome) and encapsidation (nsP1 protein). In our experiments, Dg and Dwt could thus be considered the detectable tip of the iceberg, while the vast majority of DIs unable to accumulate and propagate to such high numbers could still impair full-length virus replication.

Of note, defective interference via direct inhibition of full-length virus replication could be an effect more readily detected in BHK-21 cells, which lack the type I interferon response. If the overproduction of DIs by RNA virus mutators is a conserved phenotype *in vivo*, mutator viability may be affected both by direct interference of replication and activation of innate immunity by DI particles, as previously suggested (21). While Dg and Dwt sequences did not match previously published SINV DI sequences (31–34), we believe that discrepancies in the experimental setup can account for this difference. While early studies on SINV DIs analyzed samples passaged numerous times (30 to 50) at extremely high MOIs (>50), a setup that favors the accumulation of nonphysiological DIs, we isolated Dg and Dwt after only 24 h of infection at an MOI of 25. Furthermore, Dg and Dwt fall in a well-characterized category of DIs with large internal deletions and reinforce previous observations showing that CAA and GAA sequences with two to five adenosine residues are found in the neighborhood of the breakpoints (35, 36). To our surprise, breakpoints linking 5′ and 3′ ends of the genomes in Dg and Dwt were remarkably conserved. As DI formation primarily occurs as an accident of transcription when the replication complex jumps from one part of the genome to another, we hypothesize that strong RNA secondary structures present in these areas provoke or facilitate the RdRp jump (25, 36). While the genomes of Old World alphaviruses are bioinformatically predicted to be largely unstructured, certain regions of SINV genomes bear strong secondary structures involved in processes such as encapsidation (37, 38). However, RNA secondary structure prediction using the mfold software program did not yield any structure of interest when it was applied to the genomic regions around Dg and Dwt breakpoints (data not shown).

The high recombination rates of RNA viruses might be beneficial for viral evolution. Indeed, they are thought to favor both the accumulation of advantageous mutations on the same genome or the rescue of virus from two deleteriously mutated genomes (25). However, viral recombination might also be a consequence of genome organization, another parameter that is under selective pressure. Regardless, our data show that an increased recombination rate is coupled with a considerable accumulation of defective genome species in SINV. Because defective genomes are able to interfere with viral replication and are also potent type I interferon inducers, too high of a recombination rate could be detrimental for the virus. Recombination rate might thus be, like fidelity, a parameter that is fine-tuned under selective pressure, which affects viral fitness when it deviates from its evolutionarily optimized value. Further studies in mice and insects would provide new insights in the relationship between recombination, defective genome production, and *in vivo* attenuation.

Fidelity variants are considered interesting candidates for live attenuated vaccines due to their attenuation *in vivo* (39). Attenuation is attributed to the increase in mutation rate and the accumulation of lethal and deleterious mutations, a hypothesis strongly supported by experiments involving lethal mutagenesis using base analogs (8). However, our results demonstrate that higher recombination rates and a larger production of DI particles are also characteristics of mutator virus infection, raising the possibility that these events also contribute to attenuation.

Importantly, DIs are implicated in the preferential activation of type I interferon through the binding of cytosolic RNA sensors RIG-I and MDA5, as well as in the maturation of dendritic cells (21–24). Because of their high immunogenicity, they have been proposed as potential vaccine adjuvants (35, 40). Consequently, if RdRp mutators retain higher recombination rates and DI overproduction *in vivo*, they might have similar effects on type I interferon activation. In the future, understanding the role of each parameter (increased mutation rate and increased DI production) in the RdRp mutator phenotype would help define the contribution of each component in the *in vivo* attenuation. It remains to be shown whether lowering RdRp fidelity and increasing recombination rates/DI production are always coupled or whether some RdRp mutations can target only one of these properties. In either case, using mutator variants that generate excessive amounts of DIs could be a new way to engineer live attenuated vaccine strains that encode their own adjuvant effect.
